# Metabolic profiling, *in-situ* spatial distribution, and biosynthetic pathway of functional metabolites in *Dendrobium nobile* stem revealed by combining UPLC-QTOF-MS with MALDI-TOF-MSI

**DOI:** 10.3389/fpls.2022.1125872

**Published:** 2023-01-12

**Authors:** Qingling Liu, Yuan Huang, Chu Linghu, Jianfen Xiao, Ronghui Gu

**Affiliations:** ^1^ Key Laboratory of Plant Resource Conservation and Germplasm Innovation in Mountainous Region (Ministry of Education), Guizhou University, Guiyang, China; ^2^ College of Life Sciences, Guizhou University, Guiyang, China; ^3^ College of Pharmacy, Guizhou University of Traditional Chinese Medicine, Guiyang, China; ^4^ School of Liquor and Food Engineering, Guizhou University, Guiyang, China; ^5^ National & Local Joint Engineering Research Center for the Exploitation of Homology Resources of Medicine and Food, Guizhou University, Guiyang, China

**Keywords:** *Dendrobium nobile*, spatial imaging, UPLC-QTOF-MS, MALDI-MSI, dendrobine, sesquiterpenes, biosynthetic pathway

## Abstract

The stem of *Dendrobium nobile* Lindl. (Orchidaceae), called “*Shihu*” in traditional Chinese medicine, is a well-known medicinal and edible plant material in China. It is used as an antipyretic, analgesic, and tonic to nourish the stomach and *Yin* (i.e., to improve the production of body fluids). These therapeutic properties are attributed to its alkaloids, sesquiterpenoids, bibenzyls, fluorenones, and phenanthrenes. However, a comprehensive understanding of these metabolites and their spatial distribution in stems is lacking. In this study, ultra-performance liquid chromatography/quadrupole time-of-flight mass spectrometry (UPLC-QTOF-MS) was performed to obtain detailed metabolites information about *D. nobile* stems. Then, the spatial distributions of diverse metabolites, including alkaloids and sesquiterpenoids, were characterized and visualized by matrix-assisted laser desorption/ionization time-of-flight mass spectrometry imaging (MALDI-TOF-MSI). Based on the spatial and metabolic profiling data, sesquiterpene alkaloid dendrobine was chosen for the exhaustive study of a biosynthetic pathway in *D. nobile*. This is the first report on mass spectrometry imaging for *Dendrobium* species. As a result, critical bioactive metabolites such as 11 alkaloids, 10 sesquiterpenes, and 13 other metabolites were putatively identified and relatively quantified. The identified alkaloids were distributed in the parenchyma or vascular bundle, and sesquiterpenes were present in all regions of the stem with higher abundance in the vascular bundle and cuticle, or in the cuticle and epidermis. The biosynthetic pathway and accumulation pattern of dendrobine in *D. nobile* stem were also proposed. Our findings not only provided a critical methodology for the thorough understanding of physiological changes in metabolites and precise utilization of *D. nobile* stem, but also displayed an effective strategy for insight into the biosynthesis of bioactive metabolites in plants.

## 1 Introduction

*Dendrobium nobile* Lindl. is a prized medicinal, ornamental, and edible plant species from the genus *Dendrobium* in Orchidaceae family. The stems of *D. nobile*, *D. chrysotoxum*, *D. fimbriatum*, or *D. huoshanense* are called *Shihu* (石斛) in the *Chinese Pharmacopoeia* for nourishing *Yin* (*Yin* refers to body fluids. Nourishing *Yin*, an important term in traditional Chinese medicine (TCM), refers to improve the production of body fluids), nourishing the lung and stomach, strengthening the body, brightening the eyes, relieving coughs, and clearing heat (Nie et al., 2020). The medicinal utilization of *Shihu* in China can trace back to 2000 years ago following the records in Shennong’s Classic of Material Medica (*Shen Nong Ben Cao Jing*). The detailed document of *Shihu* was found in Compendium of Materia Medica (*Ben Cao Gang Mu*) (1590 AD) (Mou et al., 2021). Among these above-mentioned species, *D. nobile* is regarded as the base fundamental species of *Shihu*, which annual production nowadays exceeds 4.5 million kg in China (Teoh, 2019).

Due to the crucial medicinal values of *D. nobile* stem in TCM, its phytochemistry and modern pharmacology have attracted increasing attention. Some review papers have summarized that the main bioactive constituents of *D. nobile* stem were alkaloids, sesquiterpenes, dibenzyls, phenanthrenes, and polysaccharides, and the important pharmacological effects include anti-tumor, anti-oxidation, anti-aging, anti-inflammation, improving immunity, anti-fatigue, neuroprotection, and protecting the liver and kidney (He et al., 2020; Nie et al., 2020; Linghu et al., 2021; Wang et al., 2022). Among these constituents, alkaloids have caught researcher’s special concerns because it was the earliest identified classification of bioactive compounds in *Dendrobium* and responsible for numerous pharmacological effects (Chen and Chen, 1935). More importantly, dendrobine, the first active alkaloid isolated from *D. nobile* and a major ingredient in its stem, has drawn attention due to its wide applications for health benefits, such as analgesic, antipyretic, antiviral, and antihyperlipidaemic effects (Li R, et al., 2017; Gong et al., 2021). In addition, sesquiterpenes from *D. nobile* stem not only showed relatively high content but also acted as vital pharmacological activity, especially providing the most of precursors for the biosynthesis of dendrobine-type alkaloid in *D. nobile* stem (Gong et al., 2021; Mou et al., 2021). However, the comprehensive qualitative and spatial distribution of alkaloids and sesquiterpenes in mature *D. nobile* stem are still lacking.

Analyzing the metabolic profiling and spatial distribution of important metabolites in mature *D. nobile* stem is critical for the thoroughly understanding of its physiological changes and precise utilization of the prized plant resource. Recently, metabolomics has developed rapidly and applied widely to profile plant metabolites and study plant physiology (Šimura et al., 2018). In particular, the application of UPLC-QTOF-MS improved the resolution, sensitivity, and throughput for metabolites detection (Gu et al., 2019), which made the metabolomics studies possible to probe greater amounts of metabolic information for complex biological mixtures and discover previously unidentified metabolites (Tong et al., 2022). For the studies on metabolite spatial distribution, mass spectrometry imaging (MSI) has been developed as a powerful *in-situ* analysis technique to visualize the spatial distribution of metabolites in complex biological mixtures (He et al., 2019). Among MSI technologies, the matrix-assisted laser desorption/ionization mass spectrometry imaging (MALDI-MSI) is the most frequently used because of the suitable spatial resolution of imaging (3-100 *μ*m) and the wide measurable mass range with soft ionization type (Qin et al., 2018). MALDI-MSI has been proven successfully in the visualization of metabolites in plant tissues. For instance, it has been carried out to analyze the cyanogenic glucoside in sorghum (Montini et al., 2020), lipids in cottonseeds (Liu et al., 2021), and alkaloids in areca nut (Wu et al., 2022).

In this study, we focused on the metabolic profiles and their relative content in mature *D. nobile* stem, especially the spatial distribution of alkaloids and sesquiterpenes. Moreover, the accumulation pattern and biosynthesis of sesquiterpene alkaloid dendrobine in mature *D. nobile* stem were also proposed. This work reported the use of MALDI-MSI to demonstrate the distribution of metabolites in mature *D. nobile* stem for the first time, particularly in alkaloids and sesquiterpenes. The findings showed that the combination of UPLC-QTOF-MS and MALDI-MSI was a superior methodology with both sensitive and visualizable, providing a deep insight into the metabolic profiling and *in situ* spatial distribution of some special and crucial bioactive metabolites in medicinal and edible plants.

## 2 Material and methods

### 2.1 Plant material

*Dendrobium nobile* was grown on the imitation wild cultivation base at Wanglong in Chishui City, Guizhou province. Based on the growth cycle of *D. nobile*, the suitable harvest time of *D. nobile* stem was between September and October in Guizhou. Three-year-old mature stems of *D. nobile* were collected in October 2020. Immediately after harvesting, the samples were put in dry ice for transportation, and stored in a freezer at -80°C until used.

### 2.2 Chemicals

The solvents, including LC/MS-grade water, acetonitrile, methanol, formic acid, acetic acid, and trifluoroacetic acid, were bought from Sigma-Aldrich (Saint Louis, MO, USA). 2,5-dihydroxybenzoic acid (DHB), α-cyano-4-hydroxycinnamic acid (CHCA), and 2‐mercaptobenzothiazole (2-MBT) were purchased from J&K Scientific (Beijing, China). Dendrobine standard was purchased from Yuanye Shengwu (Shanghai, China).

### 2.3 Sample preparation for UPLC-QTOF-MS analysis

The fresh stem was crushed manually on ice. The crushed sample (1g) was extracted with 10 mL of 80% methanol (v/v) followed by an ultrasonic bath for 30 min at room temperature and then centrifugation (6000 r/min) for 15 min using a Cence H1850 centrifuge (Changsha, China). The supernatant was transferred, and the extraction was repeated twice. The combined supernatant was evaporated under nitrogen flow at room temperature to obtain extracts and kept at -20 °C. Prior to UPLC-QTOF-MS analysis, the extract was dissolved in 80% methanol (v/v) to a concentration of 1 mg/mL and then subjected to centrifugation (15000 r/min). The supernatant (100 μL) was added to the sample vial with a 250 μL insert pipe for analysis.

### 2.4 Sample preparation for MALDI-MSI analysis

The preparation of *D. nobile* stems sections were based on previously reported methods (He et al., 2019) with few modification. Briefly, fresh stems were stored at -80 °C until use; the stems were cryo-sectioned into 20-μm-thick slices at -20 °C on a freezing microtome (Leica CM1860, Germany) and then were immediately adhered to indium tin oxide (ITO) glass microscope slides. A matrix solution containing 2-MBT (12 mg/mL) in acetonitrile/ddH_2_O/TFA (80:20:0.2, v/v/v) was prepared and sprayed to the surfaces of stem sections by air-brush sprayer *via* five cycles (5 s spraying, 60 s drying). After air-drying in a fume hood, an extra matrix spray was conducted on the same stem sections for 40 cycles. The optical images of the stem section were obtained using a Photo Scanner (Epson Perfection V550). The standard histological optical images were gained by Hematoxylin and eosin (H&E) staining following a previous procedure (Casadonte and Caprioli, 2011).

### 2.5 UPLC-QTOF-MS and MALDI-MSI analysis

The UPLC-QTOF-MS analysis was executed with an ACQUITY UPLC coupled to Xevo G2 QTOF mass spectrometer system (Waters, Milford, MA, USA). The UPLC system was equipped with ACQUITY UPLC BEH C18 column (2.1×50 mm, I.D. 1.7 μm), autosampler, binary pump, and column compartment. The mobile phase consisted of water containing 0.1% formic acid (A) and acetonitrile containing 0.1% formic acid (B). The gradient elution procedure was as follows: 0-1.0 min, 3-13% B; 1.0-2.5 min, 13-25% B; 2.5-4.0 min, 25-40% B; 4.0-8.0 min, 40-60% B; 8.0-8.5 min, 60-97% B; 8.5-11.0 min, 97% B; 11.0-13.5 min, 97-3% B, 13.5-15.0 min, 3% B. The column temperature was 40°C, the flow rate was 0.5 mL/min, and the injection volume was 1 μL.

Mass spectrometry was obtained from both positive and negative ionization modes with scan time (1 s) and scan mass range (50-1500 Da). The capillary voltages were 3.0 kV (positive mode) and 2.5 kV (negative mode), and the sample cone voltage was 30 V. The desolvation temperature and ion source temperature were 400°C and 110°C, respectively. Nitrogen was used as the carrier gas with an 800 L/h flow rate for desolvation gas and a 50 L/h flow rate for sample cone gas. The low collision energy was 6 eV, and the high collision energy was changed repeatedly from 20 eV to 60 eV. Leucine-enkephalin (1 μg/mL) was used as Lock-Mass solution during data collection. The UPLC-QTOF-MS system was controlled by MassLynx 4.2 software (Waters, Milford, MA, USA).

MALDI-MSI was carried out on Bruker AutoFlex Speed MALDI TOF mass spectrometer (Bruker Daltonics, Germany) equipped with a 2000 Hz solid-state Smartbeam Nd: YAG UV laser (355 nm, Azura Laser AG). The detected mass ranges from 120 Da to 500 Da in positive-ion mode. Profiling data of MALDI-MS were obtained from 5000 laser shots accumulation, and each scan accumulated 500 laser shots. The images were acquired using Bruker’s *FlexImaging* v. 4.1. Moreover, serine ([M + H]^+^, m/z 106.0498), L-glutamic ([M + H]^+^, m/z 148.0604), and proline ([M + H]^+^, m/z 116.0706) were used for external mass calibration. 2-MBT ([M + H]^+^, m/z 167.994) was used for internal mass calibration. The calibration was performed with cubic enhanced mode.

### 2.6 Processing of LC-MS data

The raw mass spectrum data with *.raw* format obtained in centroid mode were converted by *Reifycs* ABF converter software (https://www.reifycs.com/AbfConverter/) to get.*abf* format files, and furtherly imported into MS-DIAL software (Ver.4.7) for peak alignment, peak picking, normalization, deconvolution, and compound identification (Tsugawa et al., 2019). The main parameter settings were as follows: retention time range, 0-15 min; MS1 tolerance, 0.015 Da; MS2 tolerance, 0.02 Da; mass range, 50-1500 Da; smoothing level, 3 scans; minimum peak width, 5 scans; retention time tolerance, 0.05 min; identification score cut off, 80%. Adduct types as [M+H]^+^, [M+Na]^+^, [M+H-H_2_O]^+^, [2M+H]^+^, and [2M +Na]^+^ in positive-ion mode, and [M-H]^-^, [M-H-H_2_O]^-^, [M+FA-H]^-^, [2M-H]^-^, and [2M+FA-H]^-^ in negative-ion mode. Identification was performed by comparing MS, MS/MS, and retention index with MS/MS-Public-Pos/Neg database in MS-DIAL. The metabolites were regarded as potential identifications when the matching degree with the spectrum database was higher than 80%. The *.txt* file results, including sample name, metabolites, peak area, retention time, and quantitative quality, were exported after MS-DAIL process. The fragment ions of exported compounds were further confirmed by its original MS and MSe data in MassLynx with mass error<10 ppm and compared with mass data reported in related databases (SciFinder, PubMed, Mzcloud) and literatures.

### 2.7 Statistical analysis

Heatmap analysis were carried out using HeatMap illustrator tool in TBtool v1.01 software. The ion maps of detected compounds were reconstructed using Bruker *FlexImaging* 4.1 software. The general histogram analysis was performed using GraphPad Prism software (GraphPad Software, Inc., La Jolla, CA, USA) and was calculated on the base of the peak intensity of identified compounds. The data were presented as mean ± standard deviation (SD).

## 3 Results and discussion

### 3.1 Metabolic profiling of *D. nobile* stems

**Figure 1:**
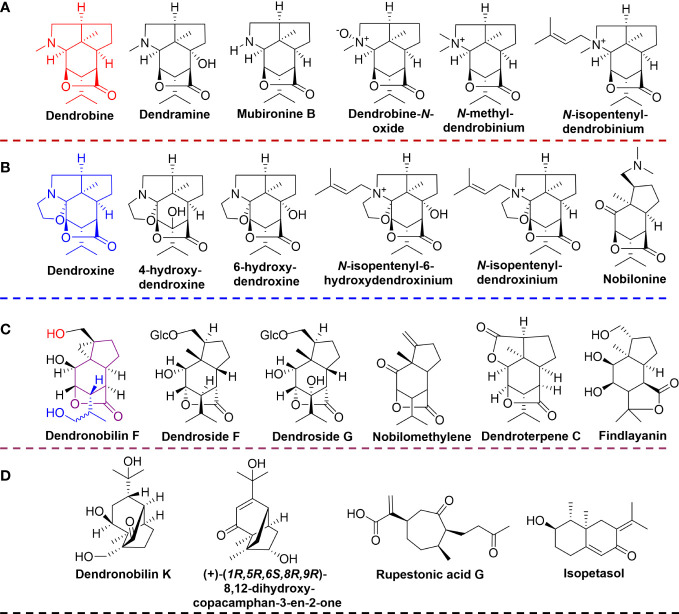
The structures of identified alkaloids and sesquiterpenes. **(A)** dendrobine-type alkaloids; **(B)** dendroxine-type alkaloids; **(C)** dendrobine-type sesquiterpenes; **(D)** other type sesquiterpenes.

Based on the established UPLC-QTOF-MS methods, the prepared extracts of *D. nobile* stems were analyzed and acquired the mass spectral data in both positive and negative ionization modes. The MS-DAIL platform and MassLynx software were used to identify and characterize the metabolites. By comparing the multi-level ion fragment information, mass spectrometry database, and related literature data, a total of 34 compounds were tentatively identified, including 11 alkaloids, 10 sesquiterpenes, 5 amino acids, 1 lignan, 1 steroid, and 6 others. Detailed information on the identifications, including compound type, name, formula, and fragments were performed in **Table S1**. The results showed alkaloids and sesquiterpenes were the main constituents in the mature *D. nobile* stem, which was accordant to the previous report that alkaloids and sesquiterpenes were the primary active constituents in the stems of *D. nobile*, especially alkaloids were representatives of the earliest identified classification of compounds from *Dendrobium* species (Xu et al., 2013; Mou et al., 2021). Based on the structural features of identified alkaloids and sesquiterpenes, we classified alkaloids as dendrobine-type alkaloids (dendrobine, dendrobine-*N*-oxide, dendramine, mubironine B, *N*-methyl-dendrobinium, and *N*-isopentenyl-dendrobinium) (**Figure 1A**), and dendroxine-type alkaloids (dendroxine, 4-hydroxy-dendroxine/6-hydroxy-dendroxine, *N*-isopentenyl-6-hydroxydendroxinium, *N*-isopentenyl-dendroxinium, and nobilonine) (**Figure 1B**). Moreover, the identified sesquiterpenes were also classified into two types, the one was dendrobine-type sesquiterpenes (findlayanin, endroterpene C, nobilomethylene, dendroside G, dendroside F, and dendronobilin F) (**Figure 1C**), and the remaining were listed as other-type (**Figure 1D**).

The alkaloids from *D. nobile* shown numerous significant pharmacological effects, including neuroprotection, hepatic lipid regulation, anti-tumor, anti-diabetes, anti-inflammatory, and anti-virus (Linghu et al., 2021). Meanwhile, the sesquiterpenes from *D. nobile* also exhibited multiple bioactivities such as anti-microbial, anti-malarial, anti-inflammatory, anti-tumor, and immunomodulatory (Gong et al., 2021).

### 3.2 Relative quantification of the identified compounds

**Figure 2:**
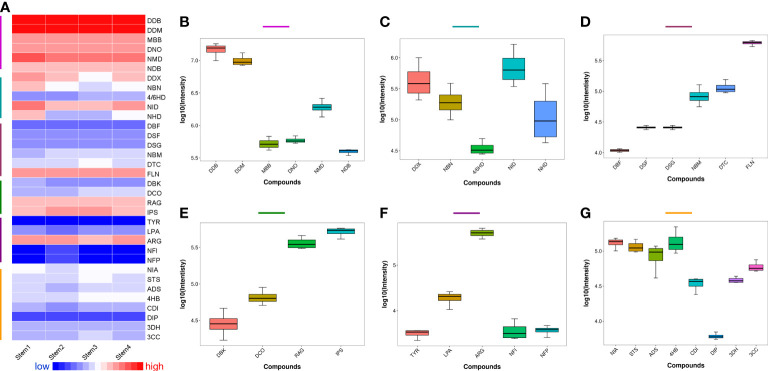
Relative content of the identified metabolites, including alkaloids and sesquiterpenes, from the mature stems of *Dendrobium nobile*. **(A)** heatmap for full view; **(B)** dendrobine-type alkaloids; **(C)** dendroxine-type alkaloids; **(D)** dendrobine-type sesquiterpenes; **(E)** other types sesquiterpenes; **(F)**: amino acid; **(G)**: other metabolites; All the full names of identified metabolites could be found in **Table S1**.

Heatmap and boxplot analysis were applied to compare the relative content of the identified different metabolites in the matured stems of *D. nobile*. As shown in **Figure 2A**, alkaloids were the most abundant metabolites in stems, especially dendrobine-type alkaloids. Sesquiterpenes presented a certain abundance, but different metabolites in sesquiterpenes were varied in relative content. Most of amino acids showed lower content in mature stems. The identified lignan (niranthin, NIA) and steroid (stigmasterol, STS) displayed higher content than most of the identified amino acids. Furtherly, the boxplot demonstrated the relative content of each identified metabolites distinctly (**Figures 2B-G**). Notably, dendrobine-type alkaloids (**Figure 2B**) had higher content than dendroxine-type alkaloids (**Figure 2C**), revealing that the alkaloid biosynthesis and accumulation were main dendrobine-type and few dendroxine-type alkaloids during the development of *D. nobile* stem. Dendrobine (DDB) has the highest accumulation than other dendrobine-type alkaloids, particularly the relative content higher than dendrobine-*N*-oxide (DNO) and dendramine (DDM) have (**Figure 2B**). Dendrobine was the characteristic and first found alkaloid from *D. nobile*, which has considered as the evaluation indicator (mass content>0.4%) for the quality control of this species by *Chinese Pharmacopoeia* (2020 edition) (Mou et al., 2021). The low accumulations of DDM, DNO, *N*-methyl-dendrobinium (NMD), and mubironine B (MBB) were gradually reduced, indicating these compounds may have little biosynthesis or may provide intermediates for the biosynthesis of DDB. The relative content of dendroxine-type alkaloids decreased in the trends following dendroxine (DDX), *N*-isopentenyl-dendroxinium (NID), nobilonine (NBN), *N*-isopentenyl-6-hydroxydendroxinium (NHD), and 4/6-hydroxy-dendroxine (4/6HD) (**Figure 2C**), revealing that NID, NBN, NHD, and 4/6HD may have little biosynthesis or decompose at mature *D. nobile* stems.

The different sesquiterpenes were also varied in the relative content of mature *D. nobile* stem (**Figures 2D-E**). For the accumulation of dendrobine-type sesquiterpenes, findlayanin (FLN) showed the highest content, followed by dendroterpene C (DTC), nobilomethylene (NBM), dendroside G (DSG), dendroside F (DSF), and dendronobilin F (DBF) (**Figure 2D**), indicating that DBF may provide the basic skeleton; and both of DSF and DSG with glycosyl group may support energy for the biosynthesis of the sesquiterpene with more complex structure (such as NBM, DTC, and FLN) during the *D. nobile* stem mature. Most dendrobine-type sesquiterpenes have been reported as intermediates for the biosynthesis of dendrobine (Gong et al., 2021). Besides, the different accumulated levels of the identified amino acid and others, including lignin (NIA) and steroid (STS), were presented in **Figures 2F, G**, respectively.

### 3.3 Visualization and spatial distribution of diverse metabolites in *D. nobile* stem

Many studies previously focused on the qualitative and quantitative of *D. nobile* metabolites, particularly in alkaloids and sesquiterpenes. However, the spatial distributions of these critical compounds in stem are still lacking, which has severely hampered the thorough understanding of *D. nobile* physiological activities and the biosynthesis of the crucial metabolites. Thus, we herein analyzed the spatial distribution of alkaloids and sesquiterpenes in *D. nobile* stem for the first time to understand the biosynthesis of metabolites.

#### 3.3.1 Optimization of sample preparation and MALDI matrix

**Figure 3:**
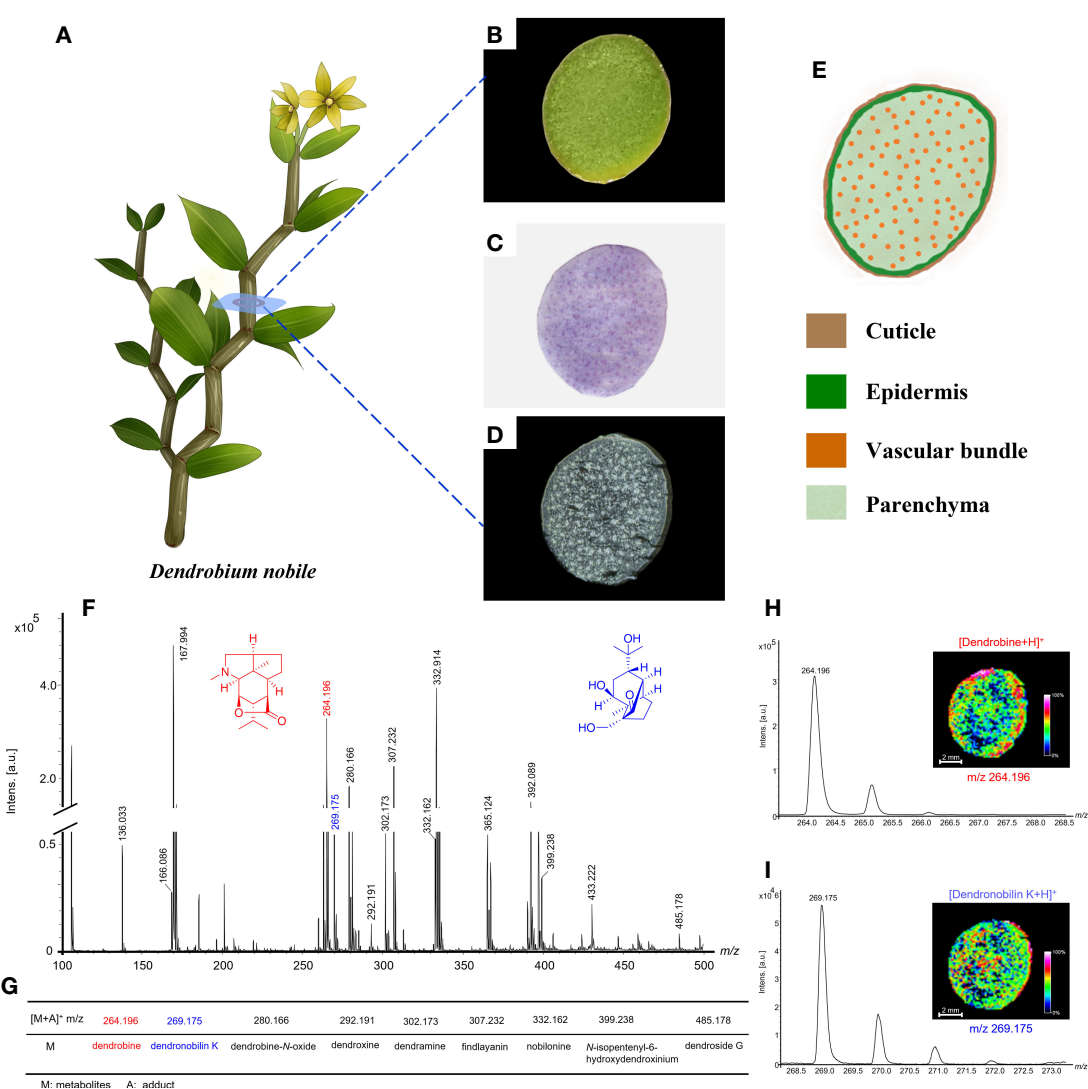
Morphology of *Dendrobium nobile* and MALDI-MS results based on optimized conditions. **(A)** Morphology of the plant; **(B)** Cross-section image of *D nobile* stem; **(C)** H&E stain image; **(D)** Frozen section; **(E)** The schematic image of cross-sections of *D nobile* stem showed the location of different tissues; **(F)** MALDI-MS results from the optimized frozen section of *D nobile* stem in positive-ion mode using 2-MBT as a matrix; **(G)** Table display of part of detected alkaloids and sesquiterpenes; **(H)** Dendrobine was used to show the optimized conditions suitable for alkaloids detection; **(I)** Dendronobilin K was used to show the optimized conditions suitable for sesquiterpenes detection.

In general, the frozen section of the samples from human and animal tissues can be prepared easily for MALDI-MSI analysis. While plant tissues difficult to prepare the frozen section due to the varied textures, waxy, and other physical characteristics. For the sample preparation of *D. nobile* stem (**Figures 3A–D**), we have investigated preparation methods, including sucrose phosphate buffer protection, chloroform washes, and slice thickness. The frozen section can present a complete cross-view without notch under the slice thickness at 20, 22, and 24 μm, but the better peak intensity performance of the mass spectrum was shown on the 20-μm-thick slices (**Figures 3B-D, F**).

Matrix was considered as a vital role in the detection of metabolites for MALDI-MSI analysis (Liu et al., 2018). 2,5-dihydroxybenzoic acid (DHB), α-cyano-4-hydroxycinnamic acid (CHCA), and 2‐mercaptobenzothiazole (2-MBT) have been widely used as MALDI matrices for the detection of low-molecular-weight metabolites (He et al., 2019). To obtain more abundant alkaloids and sesquiterpenes information, we selected DHB, CHCA, and 2-MBT as matrix to compare the performance of MALDI-MSI analysis on *D. nobile* stem. Under the same optimized conditions, 2-MBT was found with the best signal intensity distributed on the sample and the better performance of alkaloids and sesquiterpenes (**Figures 3G-I**). Thus, 2-MBT was proposed as the suitable matrix for MSI analysis in this study.

#### 3.3.2 Distribution of alkaloids in *D. nobile* mature stems

**Figure 4:**
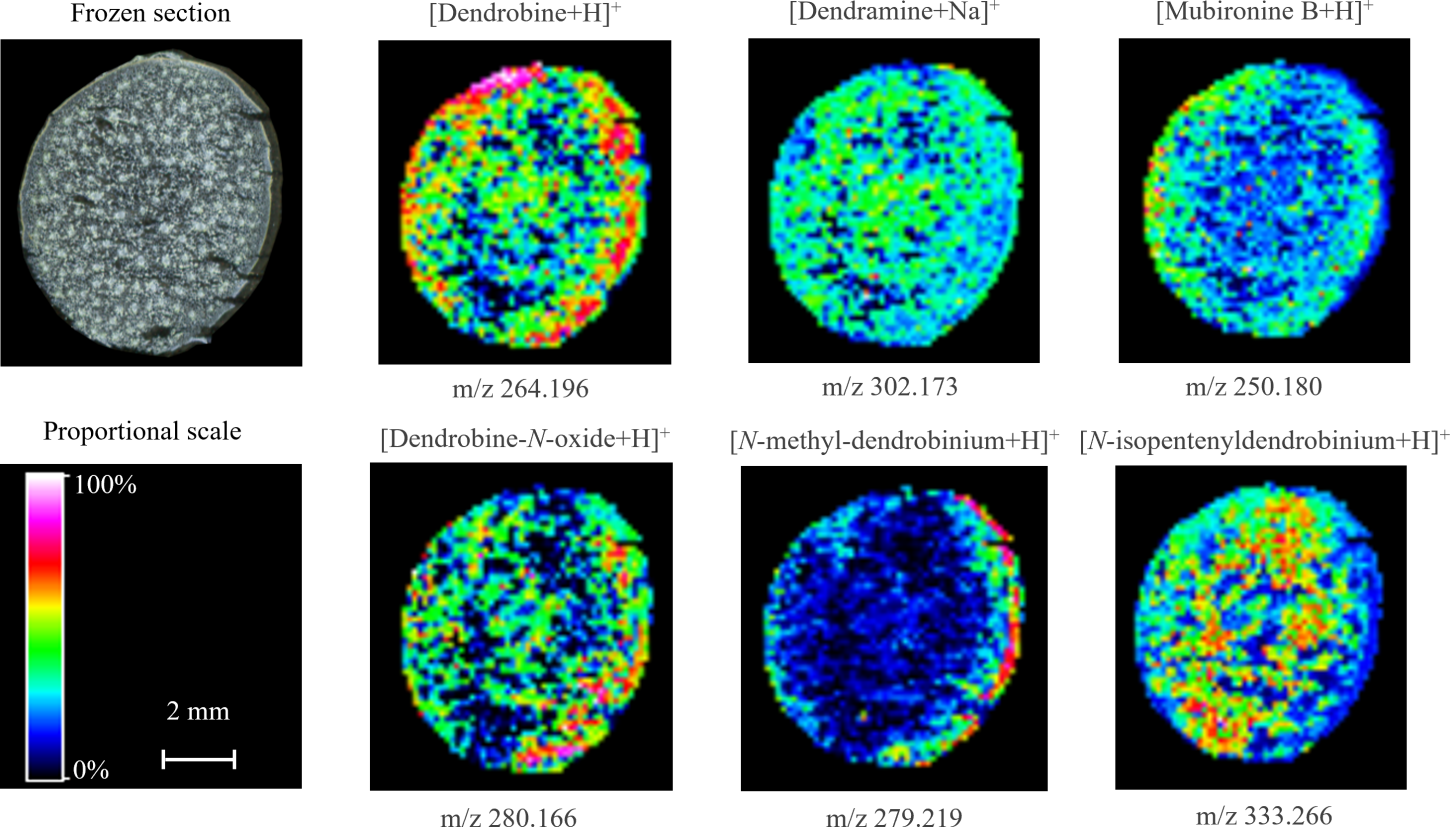
Distribution of dendrobine-type alkaloids in mature *Dendrobium nobile* stem imaged by MALDI-MSI. Heat maps were applied to present the relative distributions and contents.

As shown in **Figure 4**, the spatial distribution of dendrobine-type alkaloids was varied in the mature stem. Most of these alkaloids were distributed in the parenchyma or vascular bundle, while *N*-methyl-dendrobinium was obviously distributed in the cuticle (**Figure 3E** and **Figure 4**). As the most important bioactive metabolite in *D. nobile*, dendrobine was extremely abundant in the epidermis but also prominent in vascular bundle and little exist in parenchyma, indicating that the biosynthesis of dendrobine may occur in vascular bundle and parenchyma but transfer to epidermis for accumulation during stem maturation. The distribution of dendramine and dendrobine-*N*-oxide was found in all stem tissues, while the stronger signals were detected in vascular bundle for dendramine and in epidermis for dendrobine-*N*-oxide. However, the abundance of *N*-isopentenyl-dendrobinium and mubironine B were concentrated in vascular bundle and parenchyma and were absent in most part of cuticle.

**Figure 5:**
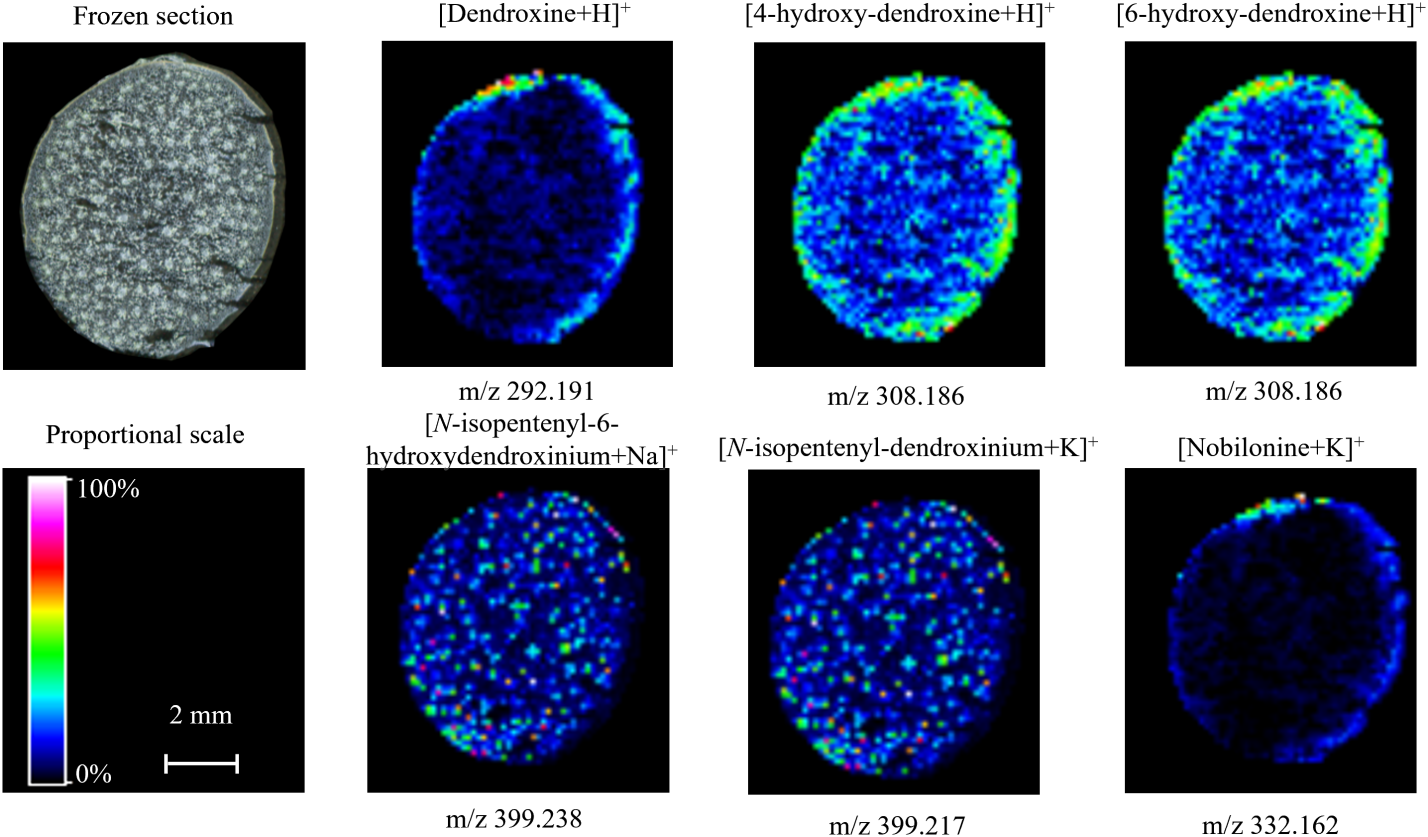
Distribution of dendroxine-type alkaloids in mature *Dendrobium nobile* stem imaged by MALDI-MSI. Heat maps were applied to present the relative distributions and contents.

The spatial distribution of identified dendroxine-type alkaloids in mature stems was almost identical, and the metabolites were obviously concentered in epidermis (**Figure 3E** and **Figure 5**). The lower signals of these metabolites indicated the low accumulation in mature *D. nobile* stem, which was consistent with their relative content detected by UPLC-QTOF-MS and speculation that dendroxine-type alkaloids may little biosynthesize. Particularly necessary to point out that 4/6-hydroxy-dendroxine was a pair of isomers, which was hard to distinguish by MALDI-MSI; thereby, the same MS image was used to show their distribution. [*N*-isopentenyl-6-hydroxydendroxinium+Na]^+^ and [*N*-isopentenyl-dendroxinium+K]^+^ also shared the same MS image for their quite similar ion mass (m/z 399.238 and 399.217) (**Figure 5**). Moreover, nobilonine could be a precursor or intermediate for biosynthesis of dendroxine- and dendrobine-type alkaloids, which was verified by its shallow signal in MS image of mature *D. nobile* stem.

#### 3.3.3 Distribution of sesquiterpenes in *D. nobile* mature stem

**Figure 6:**
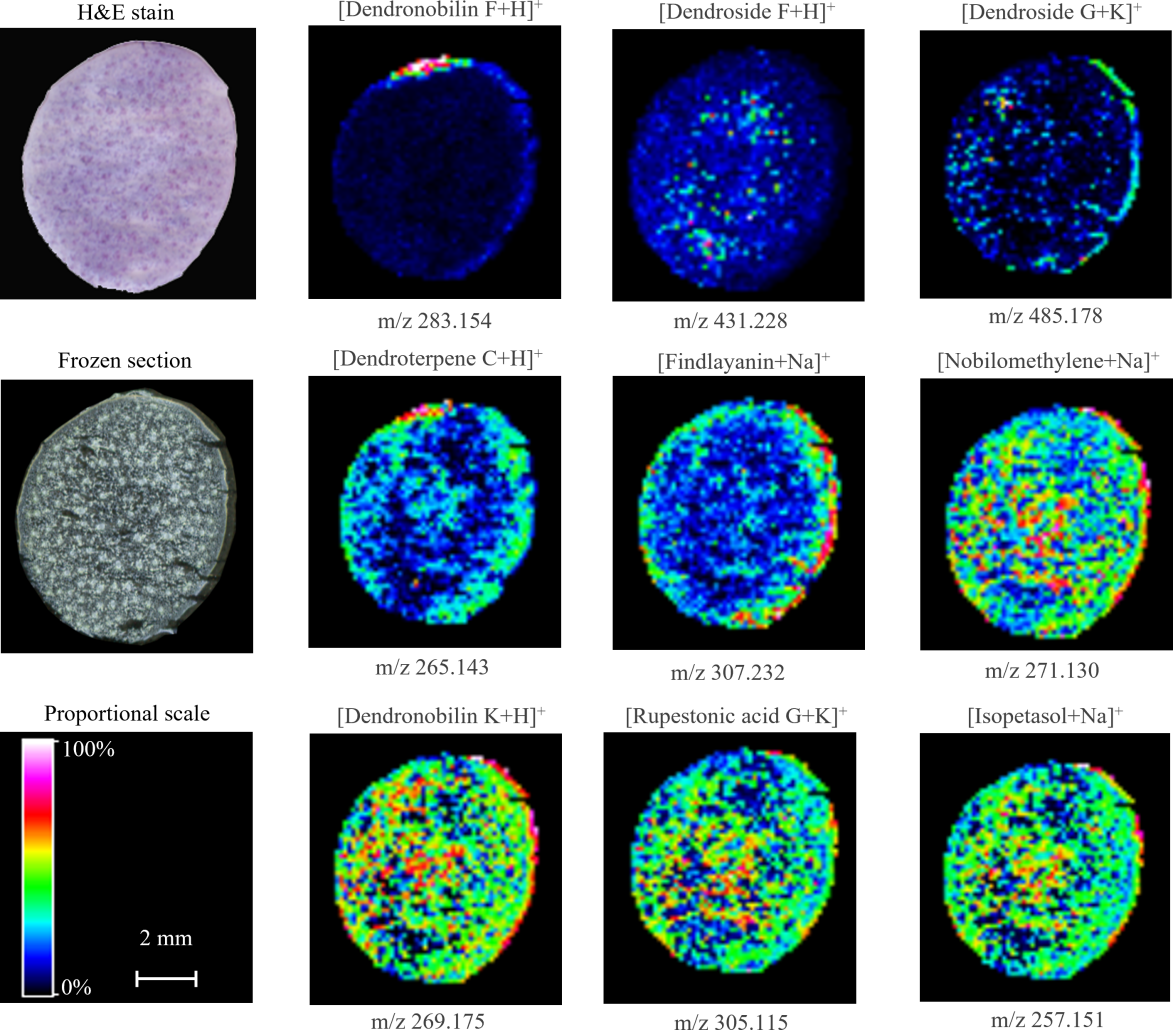
Distribution of sesquiterpenes in mature *Dendrobium nobile* stem imaged by MALDI-MSI. Heat maps were applied to present the relative distributions and contents.

We also visualized the distribution of sesquiterpenes, another kind of important medicinal substance in *D. nobile* stem, by MALDI-MSI (**Figure 6**). The cross-section imaging of mature stem showed that dendronobilin F, dendroside G, dendroterpene C, and findlayanin were concentrated in the area of the cuticle and epidermis. The distribution of nobilomethylene, dendronobilin K, rupestonic acid G, and isopetasol were present in all regions of stem with higher abundance in the vascular bundle and cuticle. Dendroside F showed a low signal in all areas of stem imaging with a relatively stronger signal in the vascular bundle. These findings revealed that most of sesquiterpene precursors were still widely distributed in the mature stem, *e.g*., nobilomethylene, dendronobilin K, rupestonic acid G, and isopetasol, while the sesquiterpene showed low abundance, *e.g*., dendronobilin F, dendroside F, dendroside G, and dendroterpene C. The spital distribution and low abundance of these metabolites demonstrated that sesquiterpenes were consumed to biosynthesize alkaloids during the maturation of *D. nobile* stem, particularly in dendrobine biosynthesis.

The spatial distribution of identified alkaloids and sesquiterpenes varied in the mature stem of *D. nobile*. The abundance of these metabolites was consistent in their relative content measured by UPLC-QTOF-MS, that is dendrobine-type alkaloids> dendroxine-type alkaloids> sesquiterpenes. Based on the accorded results from UPLC-QTOF-MS and MALDI-MSI analysis, we made a careful conclusion that sesquiterpenes were the precursors or intermediates contributing to the biosynthesis of dendrobine-type alkaloids and dendroxine-type alkaloids, which mainly accumulated in cuticle and epidermis regions, and traditional collection season of *D. nobile* (mature stem) in Guizhou was benefited for obtaining alkaloid but not for sesquiterpenes.

### 3.4 Metabolic pathway for the biosynthesis of dendrobine in *D. nobile* stem

Dendrobine, belonging to sesquiterpene alkaloids, accounted for 92.6% of *D. nobile* alkaloids (Xu et al., 2017). Moreover, dendrobine was also the first isolated bioactive alkaloid from *D. nobile* (Chen and Chen, 1935), and has been considered as the indicator ingredient for the quality evaluation of *D. nobile* stem attributing to its many important pharmacological effects (Li R et al., 2017; Song et al., 2019). Given this, the synthesis of dendrobine has attracted lots of researchers’ interest. So far, total chemical synthesis of dendrobine has been available, but the yield and purity of dendrobine still meet the challenge (Gong et al., 2021). Therefore, biosynthesis of dendrobine was prospect and primary investigation, which promoted some enzymes and genes of the biosynthetic pathway of dendrobine have been found, such as cytochrome P450 oxidase (CYP450), farnesyl diphosphate synthase (FPPS), sesquiterpene synthase (SES), 3-hydroxy-3-methylglutaryl-CoA synthase (*HMGS*), 3-hydroxy- 3-methylglutaryl-CoA reductase (*HMGR*), and mevalonate diphosphate decarboxylase (*MVD*) (Li Q et al., 2017; Gong et al., 2021; Gong et al., 2022). However, due to the enormous genome of *D. nobile* and the complex structure of dendrobine, the biosynthesis of dendrobine was still unclear.

**Figure 7:**
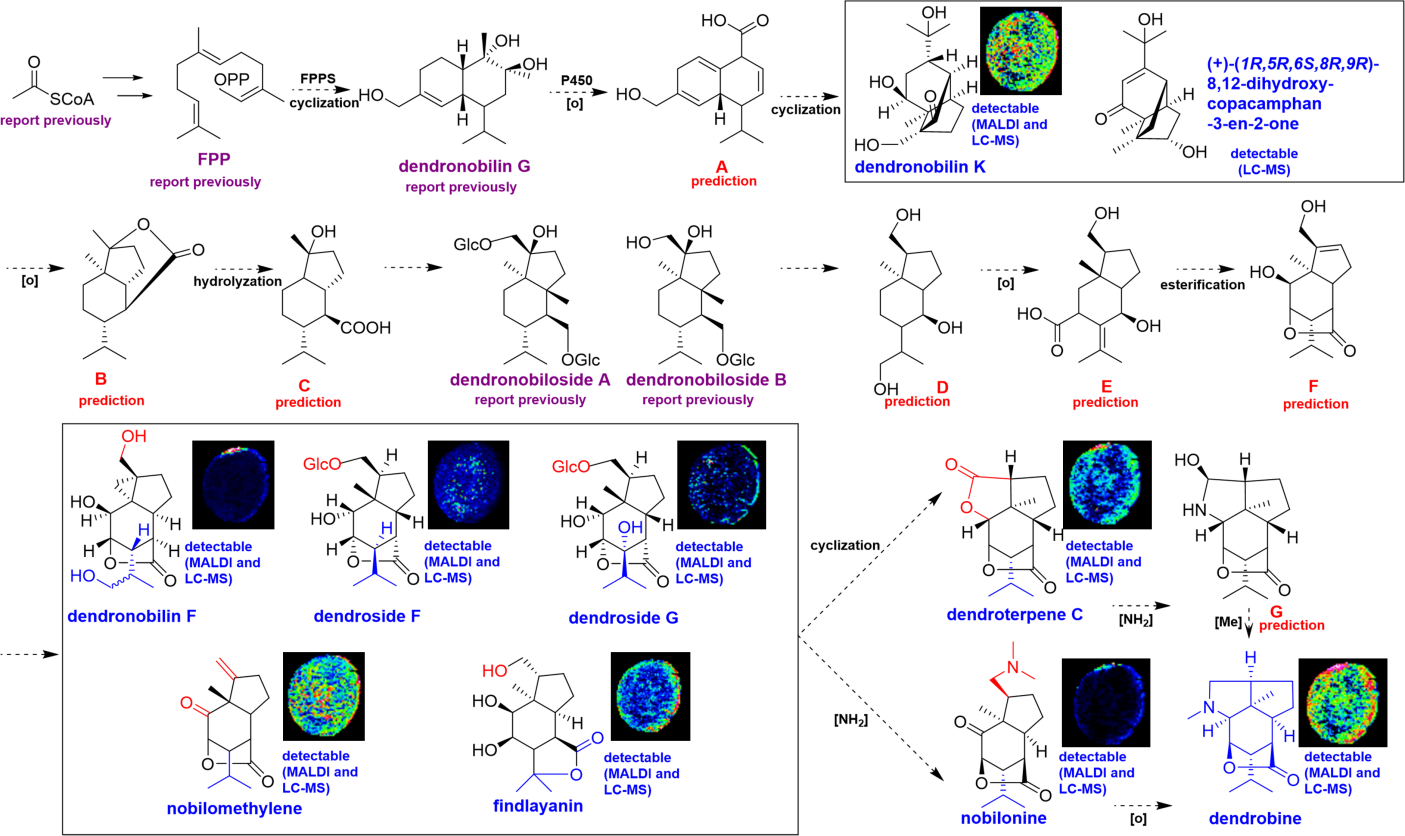
Proposed metabolic pathway for the biosynthesis of dendrobine in mature *Dendrobium nobile* stem based on the integrating analysis from UPLC-QTOF-MS and MALDI-MSI. **(A-G)** are the predicted skeleton of proposed intermediates.

This study combined the superiority of both UPLC-QTOF-MS and MALDI-MSI, high accuracy and visualizable information, to analyze the biosynthesis and accumulation of dendrobine in *D. nobile* stem. Dendrobine was a sesquiterpene alkaloid, thus, the sesquiterpenes were proposed as precursors and intermediates in dendrobine biosynthesis, which pathway was also like that of sesquiterpene due to the sesquiterpene skeleton of dendrobine. Our metabolic profiling by UPLC-QTOF-MS has revealed that, a wide variety of sesquiterpene metabolites were identified in *D. nobile* stem, whereas the abundance was low. This result corresponded to the fact that sesquiterpene was consumed for dendrobine biosynthesis during the maturation of *D. nobile* stem. **Figure 7** displayed information referring to the main compounds of the proposed dendrobine biosynthesis pathway in stems of *D. nobile* based on the data of UPLC-QTOF-MS and MALDI-MS, and previous speculation (Gong et al., 2021).

As shown in **Figure 7**, farnesyl diphosphate (FPP) was first obtained from acetyl-CoA in the mevalonate (MVA) pathway, that is, the proposed biosynthetic pathway of dendrobine was started by the catalysis of FPPS. Then FPP can form dendronobilin G through intramolecular cyclization under the action of FPPS. Dendronobilin G putatively produces intermediate A under the effects of P450 oxidoreductase. Subsequently, the intermediate A undergoes molecule rearrangement, cyclization, and Michael addition to form dendronobilin K and (+)-(1R,5R,6S,8R,9R)-8,12-dihydroxy-copacamphan-3-en-2-one. These compounds were oxidized to produce B skeleton with opened-ring and hydrolyzation, and produce C- and D-type compounds, like dendronobiloside A and dendronobiloside B. D-type intermediates undergo redox reactions furtherly to form E, and E subjected to esterification to produce intermediate F. Thus far, the skeleton of picrotoxane-type sesquiterpene (E and F) was present through the intramolecular esterification of intermediate D. We have identified and visualized five picrotoxane-type sesquiterpenes in *D. nobile* stem. These picrotoxane-type sesquiterpenes may on the one hand form dendronobilin C by cyclization, dendronobilin C was aminated to form intermediate G, and G was finally methylated to produce dendrobine. On the other hand, the sesquiterpenes may be aminated to form nobilonine, nobilonine then transfer to dendrobine by cyclization and decarboxylation. Gong et al. (2021) have speculated a similar biosynthetic pathway of dendrobine, while our work found the most of sesquiterpenes in the pathway, including their relative contents and spatial distribution, which further confirmed the biosynthetic pathway of dendrobine.

## 4 Conclusion

This work conducted a comprehensive metabolic profiling and spatial distribution of metabolites from the stem of *D. nobile* by integrating the advantages of UPLC-QTOF-MS and MALDI-TOF-MSI in high sensitivity and *in situ* visualization, respectively. The critical bioactive metabolites, such as 11 alkaloids, 10 sesquiterpenes, and 13 other metabolites, were putatively identified and relatively quantified. For the first time, *in situ* spatial distribution of metabolites in *Dendrobium* was investigated. The results revealed that most of these alkaloids were distributed in the parenchyma or vascular bundle. Meanwhile, dendrobine was highly abundant in the epidermis but also prominent in vascular bundle and little existed in parenchyma, and the identified sesquiterpenes present in all regions of stem with higher abundance in vascular bundle and cuticle, or in cuticle and epidermis. Moreover, the biosynthetic pathway and accumulation pattern of dendrobine in *D. nobile* stem were also proposed. These findings not only provided a critical methodology for the thorough understanding of physiological changes in chemical and precise utilization of *D. nobile* stem, but also displayed an effective strategy for insight into the biosynthesis of biological active plant metabolites.

## Data availability statement

The original contributions presented in the study are included in the article/**Supplementary Material**. Further inquiries can be directed to the corresponding author.

## Author contributions

RG designed and supervised the study. QL, YH, CL, and RG carried out the experiments and data analysis. QL and YH wrote the draft manuscript. JX, YH, and RG revised the manuscript. All authors contributed to the article and approved the submitted version.
