# Supplementary material for: Metabolic profiling, in-situ spatial distribution, and biosynthetic pathway of functional metabolites in Dendrobium nobile stem revealed by combining UPLC-QTOF-MS with MALDI-TOF-MSI
Source: Front Plant Sci. 2023 Jan 12;13:1125872. doi: 10.3389/fpls.2022.1125872 (PMC9878566; doi:10.3389/fpls.2022.1125872)
Supplement: Supplementary file 1 [file Table_1.docx]

Table S1. The metabolites identified from *Dendrobium nobile* stems

| No. | RT (min) | Formula | Parent ions (ppm) | Fragmental ions | Identification | Reference | Abbreviation |
| --- | --- | --- | --- | --- | --- | --- | --- |
| **Alkaloids** |  |  |  |  |  |  |  |
| **1** | 2.164 | C_16_H_25_NO_3_ | 280.1915 (-0.2) [M+H]^+^ | 318.1464, 302.1729, 252.1955, 234.1853 | Dendrobine-*N*-oxide | Zhang, et al., 2021 | DNO |
| **2** | 1.933 | C_16_H_25_NO_2_ | 264.1970 (2.5) [M+H]^+^ | 236.2002, 218.1916, 176.1432 | Dendrobine | Zhang, et al., 2021 | DDB |
| **3** | 1.181 | C_16_H_25_NO_3_ | 280.2043 (4.3) [M+H]^+^ | 262.2363, 220.1176, 192.1190 | Dendramine | Lee, et al., 1995 | DDM |
| **4** | 3.371 | C_17_H_25_NO_3_ | 292.2629 (4.6) [M+H]^+^ | 264.2104, 248.2779, 220.2280,176.1070 | Dendroxine | Okamoto, et al., 1972 | DDX |
| **5** | 3.080 | C_21_H_34_NO_2_^+^ | 332.2584 (0.3) [M]^+^ | 304.4593, 290.2115, 264.1958 | *N*-isopentenyldendrobinium | Wang, et al., 2016 | NDB |
| **6** | 2.285 | C_22_H_34_NO_4_^+^ | 376.2482 (1.8) [M]^+^ | 308.2463, 290.1751, 262.2438 | *N*-isopentenyl-6-hydroxydendroxinium | Wang, et al., 2016 | NHD |
| **7** | 7.048 | C_15_H_23_NO_2_ | 250.1816 (3.4) [M+H]^+^ | 233.1536, 206.2439, 176.1072 | Mubironine B | Wang, et al., 2016 | MBB |
| **8** | 1.323 | C_17_H_25_NO_4_ | 308.1852 (2.1) [M+H]^+^ | 346.1452, 290.1747, 280.1909, 264.1961, 262.1800 | 4-hydroxy-dendroxine/6-hydroxy-dendroxine | Okamoto, et al., 1972 | 4/6HD |
| **9** | 2.216 | C_17_H_27_NO_3_ | 294.2045 (-3.1) [M+H]^+^ | 249.1407, 221.1172, 178.0624 | Nobilonine | Wang, et al., 2010 | NBN |
| **10** | 5.882 | C_22_H_34_NO_3_^+^ | 360.2643 (-0.6) [M]^+^ | 292.1905, 264.2076, 248.1644 | *N*-isopentenyl-dendroxinium | Wang, et al., 2016 | NID |
| **11** | 1.819 | C_17_H_28_NO_2_^+^ | 278.2124 (1.4) [M]^+^ | 250.2165, 235.1567, 218.2440,190.1590 | *N*-methyl-dendrobinium | Wang, et al., 2016 | NMD |
| **Sequiterpenoids** |  |  |  |  |  |  |  |
| **12** | 1.681 | C_15_H_22_O_4_ | 267.1612 (3.3) [M+H]^+^ | 249.1500, 221.1531, 203.1440 | Rupestonic acid G | Chen, et al., 2014 | RAG |
| **13** | 1.698 | C_15_H_24_O_5_ | 285.1685 (4.1) [M+H]^+^ | 267.1573, 249.1500, 225.2429, 207.1016 | Findlayanin | Meng, et al., 2017 | FLN |
| **14** | 2.764 | C_15_H_22_O_5_ | 283.1662 (2.1) [M+H]^+^  281.1191 (-2.5) [M-H]^-^ | 265.1426, 237.1485 | Dendronobilin F | Zhang, et al., 2007 | DBF |
| **15** | 2.699 | C_15_H_24_O_4_ | 269.1740 (4.8) [M+H]^+^ | 250.2043, 233.1624, 233.1536 | Dendronobilin K | Zhang, et al., 2008 | DBK |
| **16** | 2.405 | C_15_H_22_O_3_ | 251.1652 (2.0) [M+H]^+^ | 233.1518, 205.2087 | (+)-(1*R*,5*R*,6*S*,8*R*,9*R*)-8,12-dihydroxy-copacamphan-3-en-2-one | Ma, et al., 2019 | DCO |
| **17** | 2.709 | C_21_H_34_O_9_ | 431.2267(-3.2) [M+H]^+^ | 412. 2105, 269.1740, 251.1653 | Dendroside F | Ye and Zhao, 2002 | DSF |
| **18** | 2.767 | C_15_H_20_O_4_ | 265.1426 (-4.3) [M+H]^+^ | 236.1407, 193.1443, 173.1325, 165.1253 | Dendroterpene C | Wang, et al., 2019 | DTC |
| **19** | 1.681 | C_15_H_20_O_3_ | 249.1500 (3.6) [M+H]^+^ | 231.1388, 205.1603, 133.1009, 120.0869 | Nobilomethylene | Okamoto, et al., 1972 | NBM |
| **20** | 1.854 | C_21_H_34_O_10_ | 447.2219 (-3.2) [M+H]^+^ | 469.2046, 285.1696 | Dendroside G | Zhou, et al., 2017 | DSG |
|  |  |  | 445.2061 (1.4) [M-H]^-^ | 491.2127, 283.152 |  |  |  |
| **21** | 1.835 | C_15_H_22_O_2_ | 235.1703 (2.1) [M+H]^+^ | 217.1597, 205.1574, 187.1483, 177.1272 | Isopetasol | Sugama, et al., 1983 | IPS |
| **Amino acid** |  |  |  |  |  |  |  |
| **22** | 0.457 | C_9_H_11_NO_3_ | 182.0826 (4.9) [M+H]^+^ | 165.0536, 136.0772 | Tyrosine | Si, et al., 2022 | TYR |
| **23** | 0.768 | C_9_H_11_NO_2_ | 166.0863 (-4.6) [M+H]^+^ | 147.0852, 103.0730, 91.0547, 77.0387 | Phenylalanine | Wang, et al., 2021 | LPA |
| **24** | 0.285 | C_6_H_14_N_4_O_2_ | 175.1187 (3.4) [M+H]^+^ | 158.0922 | Arginine | Song, et al., 2020 | ARG |
|  |  |  | 173.1036 (2.2) [M-H]^-^ |  |  |  |  |
| **25** | 0.567 | C_12_H_23_NO_7_ | 294.1553 (0.7) [M+H]^+^ | 276.1441, 258.1329, 146.1302 | *N*-(1-Deoxy-*β*-D-fructopyranos-1-yl)-L-isoleucine | Zhang, et al., 2022 | NFI |
| **26** | 0.785 | C_15_H_21_NO_7_ | 328.1403 (-2.4) [M+H]^+^ | 310.1301, 179.1574, 162.0523 | *N*-(1-Deoxy-*β*-D-fructopyranos-1-yl)-L-phenylalanine | Zhang, et al., 2022 | NFP |
| **Lignin** |  |  |  |  |  |  |  |
| **27** | 5.543 | C_24_H_32_O_7_ | 433.3241 (-3.1) [M+H]^+^ | 400.1880, 376.2042, 208.1294, 178.0998 | Niranthin | Singh, et al., 2022 | NIA |
| **Sterides** |  |  |  |  |  |  |  |
| **28** | 9.345 | C_29_H_48_O | 413.3783 (4.4) [M+H]^+^ | 394.3644, 383.3408, 301.2555 | Stigmasterol | Nie, et al., 2020 | STS |
| **Others** |  |  |  |  |  |  |  |
| **29** | 0.46 | C_10_H_13_N_5_O_4_ | 268.1046 (-1.6) [M+H]^+^ | 136.0629, 132.0453 | Adenosine | Xiao, et al., 2016 | ADS |
| **30** | 9.184 | C_10_H_12_O_2_ | 165.0908 (2.2) [M+H]^+^ | 146.1526, 138.0958, 122.0842 | 4-(4-Hydroxyphenyl)-2-butanone | - | 4HB |
| **31** | 3.354 | C_15_H_24_O_2_ | 237.1850 (3.1) [M+H]^+^ | 219.2274, 201.1638 | *δ*-Cadinen-12,14-diol | Xiao, et al., 2016 | CDI |
| **32** | 7.250 | C_16_H_22_O_4_ | 279.2004 (0.2) [M+H]^+^ | 301.1036, 205.0884, 149.0333 | Dibutyl phthalate | Chen, et al., 2008 | DIP |
| **33** | 2.767 | C_14_H_22_O_4_ | 255.1623 (3.6) [M+H]^+^ | 237.1472, 226.2563, 211.1739, 173.1325 | 3-hydroxy-1α,5-bis(hydroxymethyl)-5,6β-dimethyl-1,3,3α,4,6,6α-hexahydrocyclopropa[e]inden-2-one | - | 3DH |
| **34** | 1.560 | C_14_H_22_O_3_ | 239.1563 (4.1) [M+H]^+^ | 221.1531, 203.1430, 168.2463, 107.0855 | 3-hydroxy-5,5,8α-trimethyl-3,4,4α,6,7,8-hexahydronaphthalene-2-carboxylic acid | Fukuyama, et al., 1985 | 3CC |

**Reference**

Chen, Y., Li, J., Wang, L., and Liu, Y. (2008). Aromatic compounds from *Dendrobium aphyllum*. *Biochemical Systematics and Ecology, 5*(36), 458-460. doi:10.1016/j.bse.2007.11.004

Chen, Z., Wang, S., Zeng, K.-W., Cui, F.-X., Jin, H.-W., Guo, X.-Y., et al. (2014). Rupestonic acids B–G, NO inhibitory sesquiterpenoids from *Artemisia rupestris*. *Bioorganic & Medicinal Chemistry Letters, 24*(17), 4318-4322. doi: 10.1016/j.phymed.2009.05.010

Fukuyama, Y., Sato, T., Miura, I., and Asakawa, Y. (1985). Drimane-type sesqui-and norsesquiterpenoids from *Polygonum hydropiper*. *Phytochemistry, 24*(7), 1521-1524. doi: 10.1016/S0031-9422(00)81058-4

Lee, Y. H., Park, J. D., Beak, N. I., Kim, S. I., and Ahn, B. Z. (1995). In vitro and in vivo antitumoral phenanthrenes from the aerial parts of *Dendrobium nobile*. *Planta Medica, 61*(02), 178-180. doi: 10.1055/s-2006-958043

Ma, C., Meng, C.-W., Zhou, Q.-M., Peng, C., Liu, F., Zhang, J.-W., et al. (2019). New sesquiterpenoids from the stems of *Dendrobium nobile* and their neuroprotective activities. *Fitoterapia, 138*, 104351. doi: 10.1016/j.fitote.2019.104351

Meng, C.-W., He, Y.-L., Peng, C., Ding, X.-J., Guo, L., and Xiong, L. (2017). Picrotoxane sesquiterpenoids from the stems of *Dendrobium nobile* and their absolute configurations and angiogenesis effect. *Fitoterapia, 121*, 206-211. doi: 10.1016/j.fitote.2017.07.017

Nie, X., Chen, Y., Li, W., and Lu, Y. (2020). Anti-aging properties of *Dendrobium nobile* Lindl.: From molecular mechanisms to potential treatments. *Journal of Ethnopharmacology, 257*, 112839. doi: 10.1016/j.jep.2020.112839

Okamoto, T., Natsume, M., Onaka, T., Uchimaru, F., and Shimizu, M. (1972). Further studies on the alkaloidal constituents of *Dendrobium nobile* (Orchidaceae)-structure determination of 4-hydroxy-dendroxine and nobilomethylene. *Chemical and Pharmaceutical Bulletin, 20*(2), 418-421. doi: 10.1248/cpb.20.418

Si, C., Zeng, D., Yu, Z., da Silva, J. A. T., Duan, J., He, C., et al. (2022). Transcriptomic and metabolomic analyses reveal the main metabolites in *Dendrobium officinale* leaves during the harvesting period. *Plant Physiology and Biochemistry, 190*, 24-34. doi: 10.1016/j.plaphy.2022.08.026

Singh, V. K., Trivedi, S., Shukla, K., and Singh, M. (2022). Phytochemical assessment from callus and shoot cultures of a potential medicinal herb: *Phyllanthus Amarus* (Schum. & Thonn). *Plant Archives, 22*(1), 109-114. doi: 10.51470/PLANTARCHIVES.2022.v22.no1.017.

Song, C., Jiao, C., Jin, Q., Chen, C., Cai, Y., and Lin, Y. (2020). Metabolomics analysis of nitrogen-containing metabolites between two Dendrobium plants. *Physiology and Molecular Biology of Plants, 26*(7), 1425-1435. doi: 10.1007/s12298-020-00822-1

Sugama, K., Hayashi, K., Nakagawa, T., Mitsuhashi, H., and Yoshida, N. (1983). Sesquiterpenoids from *Petasites fragrans*. *Phytochemistry, 22*(7), 1619-1622. doi: 10.1016/0031-9422(83)80099-5

Wang, P., Chen, X., Wang, H., Huang, S., Cai, C., Yuan, J., et al. (2019). Four new picrotoxane-type sesquiterpenes from *Dendrobium nobile* Lindl. *Frontiers in chemistry, 7*, 812. doi: 10.3389/fchem.2019.00812

Wang, Q., Gong, Q., Wu, Q., and Shi, J. (2010). Neuroprotective effects of Dendrobium alkaloids on rat cortical neurons injured by oxygen-glucose deprivation and reperfusion. *Phytomedicine, 17*(2), 108-115. doi: 10.1016/j.phymed.2009.05.010

Wang, Y.-H., Avula, B., Abe, N., Wei, F., Wang, M., Ma, S.-C., et al. (2016). Tandem mass spectrometry for structural identification of sesquiterpene alkaloids from the stems of *Dendrobium nobile* using LC-QToF. *Planta Medica, 82*(07), 662-670. doi: 10.1055/s-0042-103031

Wang, Z., Jiang, W., Liu, Y., Meng, X., Su, X., Cao, M., et al. (2021). Putative genes in alkaloid biosynthesis identified in *Dendrobium officinale* by correlating the contents of major bioactive metabolites with genes expression between Protocorm-like bodies and leaves. *BMC Genomics, 22*(1), 1-17. doi: 10.1186/s12864-021-07887-6

Xiao, S.-J., Qian, Y., Zhang, L., Tang, Y., Zhu, X., Zhou, H.-X., et al. (2016). A new sesquiterpene from *Dendrobium nobile*. *Chinese Traditional and Herbal Drugs, 47*, 2972-2974. doi:10.7501/j.issn.0253-2670.2016.17.002

Ye, Q., and Zhao, W. (2002). New alloaromadendrane, cadinene and cyclocopacamphane type sesquiterpene derivatives and bibenzyls from *Dendrobium nobile*. *Planta medica, 68*(08), 723-729. doi: 10.1055/s-2002-33786

Zhang, F., Li, B., Wen, Y., Liu, Y., Liu, R., Liu, J., et al. (2022). An integrated strategy for the comprehensive profiling of the chemical constituents of *Aspongopus Chinensis* using UPLC-QTOF-MS combined with molecular networking. *Pharmaceutical Biology, 60*(1), 1349-1364. doi: 10.1080/13880209.2022.2096078

Zhang, M.-S., Linghu, L., Wang, G., He, Y.-Q., Sun, C.-X., and Xiao, S.-J. (2021). Dendrobine-type alkaloids from *Dendrobium nobile*. *Natural Product Research*, *36*(21), 5393-5399. doi: 10.1080/14786419.2021.2019731

Zhang, X., Liu, H. W., Gao, H., Han, H. Y., Wang, N. L., Wu, H. M., et al. (2007). Nine new sesquiterpenes from *Dendrobium nobile*. *Helvetica chimica acta, 90*(12), 2386-2394. doi: 10.1002/hlca.200790245

Zhang, X., Tu, F.-J., Yu, H.-Y., Wang, N.-L., Wang, Z., and Yao, X.-S. (2008). Copacamphane, picrotoxane and cyclocopacamphane sesquiterpenes from *Dendrobium nobile*. *Chemical and Pharmaceutical Bulletin, 56*(6), 854-857. doi: 10.1248/cpb.56.854

Zhou, X.-M., Zheng, C.-J., Wu, J.-T., Chen, G.-Y., Zhang, B., and Sun, C.-G. (2017). A new phenolic glycoside from the stem of *Dendrobium nobile*. *Natural Product Research, 31*(9), 1042-1046. doi: 10.1080/14786419.2016.1266352
